# Bis(μ_2_-iso­propyl­imido-κ^2^ *N*:*N*)bis­[(η^5^-cyclo­penta­dien­yl)(ethenolato-κ*O*)titanium(IV)]

**DOI:** 10.1107/S1600536813033072

**Published:** 2013-12-14

**Authors:** Martin Haehnel, Anke Spannenberg, Uwe Rosenthal

**Affiliations:** a Leibniz-Institut für Katalyse e. V. an der Universität Rostock, Albert-Einstein-Strasse 29a, 18059 Rostock, Germany

## Abstract

The title dinuclear half-sandwich complex, [CpTi(OCH=CH_2_)(μ_2_-N-*i*Pr)]_2_ (Cp = cyclo­penta­dien­yl; *i*Pr = isopropyl), was ob­tained from the reaction of Cp_2_TiCl_2_, *n*-butyl­lithium and iso­propyl­amine in tetra­hydro­furan. Each Ti^IV^ atom is coordinated by one Cp ligand, one vin­yloxy unit and two bridging imido groups in a strongly distorted tetra­hedral geometry. There are two half mol­ecules in the asymmetric unit, such that whole mol­ecules being generated by inversion symmetry.

## Related literature   

For other Ti complexes with both Cp′ (Cp′ = substituted or unsubstituted Cp) ligands and an enolate unit with a terminal =CH_2_ group, see: Curtis *et al.* (1984 ▶); Veya *et al.* (1993 ▶); Beckhaus *et al.* (1994 ▶); Schwartz *et al.* (1996 ▶). For selected examples of half-sandwich CpTi complexes with μ_2_-bridging imido ligands, see: Vroegop *et al.* (1983 ▶); Grigsby *et al.* (1996 ▶); Ascenso *et al.* (2001 ▶); Tsurugi *et al.* (2011 ▶).
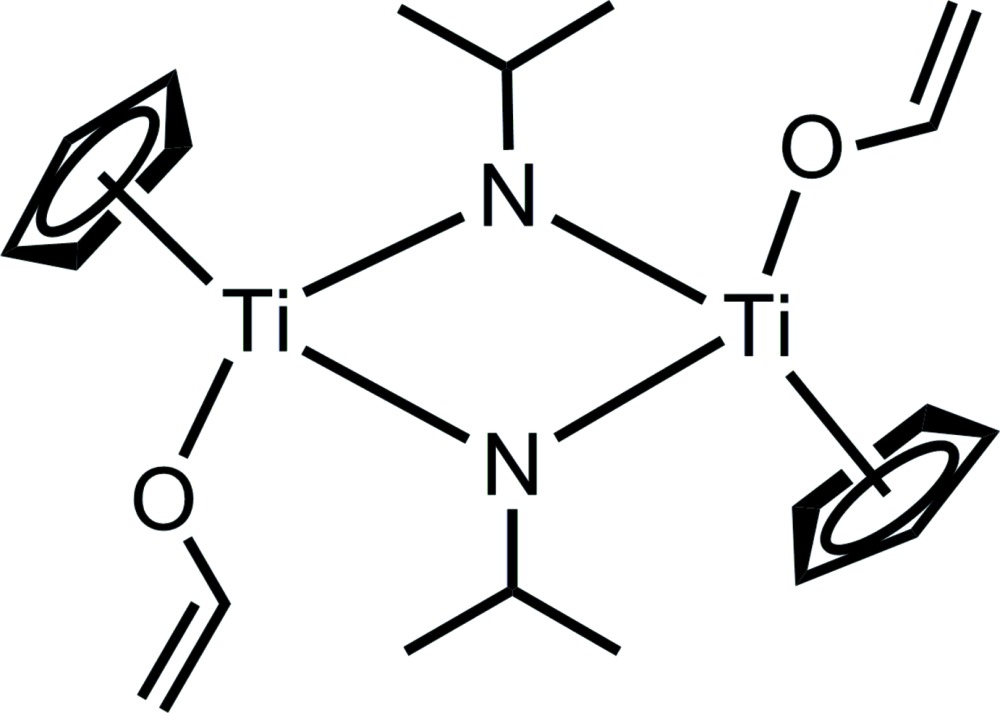


## Experimental   

### 

#### Crystal data  

[Ti_2_(C_5_H_5_)_2_(C_3_H_7_N)_2_(C_2_H_3_O)_2_]
*M* *_r_* = 426.26
Monoclinic, 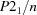

*a* = 13.8746 (4) Å
*b* = 9.7484 (2) Å
*c* = 16.3264 (4) Å
β = 106.593 (2)°
*V* = 2116.27 (9) Å^3^
*Z* = 4
Mo *K*α radiation
μ = 0.77 mm^−1^
*T* = 150 K
0.50 × 0.40 × 0.25 mm

#### Data collection  

Stoe IPDS II diffractometer
32869 measured reflections
4625 independent reflections
4211 reflections with *I* > 2σ(*I*)
*R*_int_ = 0.031

#### Refinement  

*R*[*F* ^2^ > 2σ(*F* ^2^)] = 0.024
*wR*(*F* ^2^) = 0.069
*S* = 1.05
4625 reflections
239 parameters
H-atom parameters constrained
Δρ_max_ = 0.41 e Å^−3^
Δρ_min_ = −0.37 e Å^−3^

### 

Data collection: *X-AREA* (Stoe & Cie, 2005 ▶); cell refinement: *X-AREA*; data reduction: *X-AREA*; program(s) used to solve structure: *SHELXS97* (Sheldrick, 2008 ▶); program(s) used to refine structure: *SHELXL97* (Sheldrick, 2008 ▶); molecular graphics: *XP* in *SHELXTL* (Sheldrick, 2008 ▶); software used to prepare material for publication: *SHELXTL*.

## Supplementary Material

Crystal structure: contains datablock(s) I, Global. DOI: 10.1107/S1600536813033072/pk2508sup1.cif

Structure factors: contains datablock(s) I. DOI: 10.1107/S1600536813033072/pk2508Isup2.hkl

Additional supporting information:  crystallographic information; 3D view; checkCIF report

## Figures and Tables

**Fig. 1.**
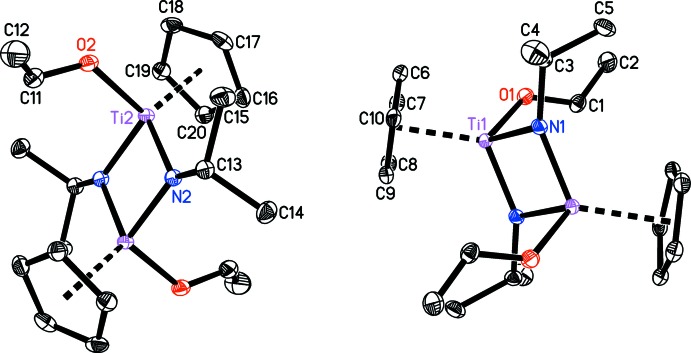
Molecular structure of the title compound in the crystal (the asymmetric unit contains two half molecules; operators for generating equivalent atoms: -x + 2, -y + 1, -z and -x + 1, -y, -z). Hydrogen atoms are omitted for clarity. Displacement ellipsoids are drawn at the 30% probability level.

## supplementary crystallographic information

### 1. Comment

The reaction of Cp_2_TiCl_2_, *n*-butyllithium and isopropylamine in THF was investigated to synthesize the new titanocene imido compound Cp_2_Ti=N-*i*Pr. However, the excess of *n*-butyllithium led to THF cleavage, involving the formation of vinyloxy species. Substitution of one of the Cp ligands by the vinyloxy ligand was observed, together with the imido unit bridging over two titanium centers, thus forming a dinuclear complex. The asymmetric unit contains two half molecules of the title compound. Each titanium center is coordinated by one Cp ligand, one vinyloxy unit and two bridging imido groups (Fig. 1). The geometry at the titanium centers is strongly distorted tetrahedral. The largest deviation from the ideal tetrahedral angle is observed for N2—Ti2—N2A with 85.84 (4)°. The central four-membered metallacycles Ti1, N1, Ti1A, N1A and Ti2, N2, Ti2A, N2A are planar by virtue of the inversion symmetry.

### 2. Experimental

To a stirred solution of isopropylamine (0.3 ml, 3.5 mmol) in 20 ml of THF was added a solution of *n*-butyllithium (1.6 *M*, 5.0 ml) in *n*-hexane at room temperature. After stirring fo 16 h, the reaction mixture was slowly poured into a suspension of Cp_2_TiCl_2_ (872 mg, 3.5 mmol) in 15 ml of THF. Immediately, the colour turned dark brown. After additional stirring for 20 h, all volatiles were removed in vacuum and the dark brown residue was suspended in 40 ml of diethylether and filtered. The dark brown solution was then concentrated to 5 ml and stored at -78 °C for 3 days to give dark red crystals of the title compound, suitable for X-ray analysis, which were filtered, washed with cold toluene and dried in vacuum (yield: 256 mg, 34%)

### 3. Refinement

H atoms were placed in idealized positions with d(C—H) = 0.95 - 1.00 Å (CH), 0.95 Å (CH_2_) and 0.98 Å (CH_3_) and refined using a riding model with *U*_iso_(H) fixed at 1.2 *U*_eq_(C) for CH, CH_2_ and 1.5 *U*_eq_(C) for CH_3_.

### Crystal data

**Table:**

[Ti_2_(C_5_H_5_)_2_(C_3_H_7_N)_2_(C_2_H_3_O)_2_]	*F*(000) = 896
*M**_r_* = 426.26	*D*_x_ = 1.338 Mg m^−^^3^
Monoclinic, *P*2_1_/*n*	Mo *K*α radiation, λ = 0.71073 Å
*a* = 13.8746 (4) Å	Cell parameters from 4113 reflections
*b* = 9.7484 (2) Å	θ = 2.5–29.5°
*c* = 16.3264 (4) Å	µ = 0.77 mm^−^^1^
β = 106.593 (2)°	*T* = 150 K
*V* = 2116.27 (9) Å^3^	Prism, brown
*Z* = 4	0.50 × 0.40 × 0.25 mm

### Data collection

**Table:**

Stoe IPDS II diffractometer	4211 reflections with *I* > 2σ(*I*)
Radiation source: fine-focus sealed tube	*R*_int_ = 0.031
Graphite monochromator	θ_max_ = 27.0°, θ_min_ = 1.7°
ω scans	*h* = −17→17
32869 measured reflections	*k* = −12→12
4625 independent reflections	*l* = −20→20

### Refinement

**Table:**

Refinement on *F*^2^	Primary atom site location: structure-invariant direct methods
Least-squares matrix: full	Secondary atom site location: difference Fourier map
*R*[*F*^2^ > 2σ(*F*^2^)] = 0.024	Hydrogen site location: inferred from neighbouring sites
*wR*(*F*^2^) = 0.069	H-atom parameters constrained
*S* = 1.05	*w* = 1/[σ^2^(*F*_o_^2^) + (0.0485*P*)^2^ + 0.2691*P*] where *P* = (*F*_o_^2^ + 2*F*_c_^2^)/3
4625 reflections	(Δ/σ)_max_ = 0.002
239 parameters	Δρ_max_ = 0.41 e Å^−^^3^
0 restraints	Δρ_min_ = −0.37 e Å^−^^3^

### Special details

**Table:**

Geometry. All e.s.d.'s (except the e.s.d. in the dihedral angle between two l.s. planes) are estimated using the full covariance matrix. The cell e.s.d.'s are taken into account individually in the estimation of e.s.d.'s in distances, angles and torsion angles; correlations between e.s.d.'s in cell parameters are only used when they are defined by crystal symmetry. An approximate (isotropic) treatment of cell e.s.d.'s is used for estimating e.s.d.'s involving l.s. planes.
Refinement. Refinement of *F*^2^ against ALL reflections. The weighted *R*-factor *wR* and goodness of fit *S* are based on *F*^2^, conventional *R*-factors *R* are based on *F*, with *F* set to zero for negative *F*^2^. The threshold expression of *F*^2^ > σ(*F*^2^) is used only for calculating *R*-factors(gt) *etc*. and is not relevant to the choice of reflections for refinement. *R*-factors based on *F*^2^ are statistically about twice as large as those based on *F*, and *R*- factors based on ALL data will be even larger.

### Fractional atomic coordinates and isotropic or equivalent isotropic displacement parameters (Å^2^)

**Table:**

	*x*	*y*	*z*	*U*_iso_*/*U*_eq_	
C1	1.00508 (9)	0.81266 (12)	0.07526 (8)	0.0264 (2)	
H1	1.0139	0.8211	0.0199	0.032*	
C2	1.04722 (11)	0.90582 (14)	0.13237 (9)	0.0398 (3)	
H2A	1.0400	0.9008	0.1884	0.048*	
H2B	1.0849	0.9783	0.1176	0.048*	
C3	1.10447 (9)	0.42926 (12)	0.16322 (7)	0.0237 (2)	
H3	1.0485	0.4372	0.1903	0.028*	
C4	1.14785 (12)	0.28630 (14)	0.18057 (9)	0.0406 (3)	
H4A	1.0950	0.2187	0.1567	0.061*	
H4B	1.1743	0.2723	0.2424	0.061*	
H4C	1.2023	0.2753	0.1539	0.061*	
C5	1.18229 (11)	0.53813 (14)	0.20265 (8)	0.0344 (3)	
H5A	1.2402	0.5285	0.1801	0.052*	
H5B	1.2043	0.5268	0.2649	0.052*	
H5C	1.1526	0.6294	0.1884	0.052*	
C6	0.83879 (9)	0.43737 (12)	0.13292 (7)	0.0252 (2)	
H6	0.8677	0.4425	0.1931	0.030*	
C7	0.77320 (9)	0.53364 (12)	0.08185 (8)	0.0273 (2)	
H7	0.7498	0.6153	0.1015	0.033*	
C8	0.74794 (9)	0.48839 (13)	−0.00364 (8)	0.0273 (2)	
H8	0.7049	0.5344	−0.0516	0.033*	
C9	0.79764 (8)	0.36312 (12)	−0.00555 (7)	0.0249 (2)	
H9	0.7938	0.3094	−0.0549	0.030*	
C10	0.85404 (8)	0.33132 (12)	0.07872 (7)	0.0241 (2)	
H10	0.8951	0.2525	0.0961	0.029*	
C11	0.49256 (11)	−0.21964 (14)	0.14913 (8)	0.0350 (3)	
H11	0.4293	−0.2371	0.1088	0.042*	
C12	0.54472 (15)	−0.32585 (17)	0.18746 (11)	0.0544 (4)	
H12A	0.6083	−0.3125	0.2281	0.065*	
H12B	0.5188	−0.4160	0.1745	0.065*	
C13	0.69659 (8)	−0.08443 (12)	0.03329 (7)	0.0232 (2)	
H13	0.6907	−0.1851	0.0212	0.028*	
C14	0.75543 (10)	−0.02130 (15)	−0.02278 (9)	0.0331 (3)	
H14A	0.7197	−0.0378	−0.0830	0.050*	
H14B	0.8224	−0.0631	−0.0093	0.050*	
H14C	0.7622	0.0777	−0.0122	0.050*	
C15	0.75381 (10)	−0.06547 (16)	0.12745 (8)	0.0356 (3)	
H15A	0.7647	0.0326	0.1400	0.053*	
H15B	0.8189	−0.1123	0.1397	0.053*	
H15C	0.7146	−0.1045	0.1631	0.053*	
C16	0.59925 (10)	0.27006 (13)	0.09822 (8)	0.0324 (3)	
H16	0.6481	0.2876	0.0689	0.039*	
C17	0.61833 (10)	0.21581 (14)	0.18150 (8)	0.0344 (3)	
H17	0.6823	0.1899	0.2179	0.041*	
C18	0.52699 (10)	0.20677 (13)	0.20118 (7)	0.0311 (3)	
H18	0.5181	0.1745	0.2535	0.037*	
C19	0.45087 (9)	0.25346 (12)	0.13058 (8)	0.0278 (2)	
H19	0.3812	0.2576	0.1264	0.033*	
C20	0.49548 (10)	0.29353 (12)	0.06639 (8)	0.0290 (2)	
H20	0.4614	0.3297	0.0118	0.035*	
N1	1.06135 (7)	0.44839 (9)	0.07062 (6)	0.02088 (18)	
N2	0.59469 (7)	−0.02696 (9)	0.01025 (6)	0.02089 (19)	
O1	0.95030 (6)	0.70628 (8)	0.08943 (5)	0.02590 (17)	
O2	0.52192 (6)	−0.08863 (9)	0.16262 (5)	0.02749 (18)	
Ti1	0.924511 (14)	0.532856 (19)	0.036855 (12)	0.01873 (7)	
Ti2	0.520839 (14)	0.05278 (2)	0.083057 (12)	0.01907 (7)	

### Atomic displacement parameters (Å^2^)

**Table:**

	*U*^11^	*U*^22^	*U*^33^	*U*^12^	*U*^13^	*U*^23^
C1	0.0297 (6)	0.0218 (5)	0.0307 (6)	−0.0005 (4)	0.0136 (5)	0.0008 (4)
C2	0.0497 (8)	0.0313 (7)	0.0409 (7)	−0.0158 (6)	0.0170 (6)	−0.0048 (5)
C3	0.0243 (5)	0.0265 (5)	0.0189 (5)	0.0013 (4)	0.0038 (4)	0.0020 (4)
C4	0.0452 (8)	0.0312 (7)	0.0392 (7)	0.0105 (6)	0.0020 (6)	0.0085 (5)
C5	0.0363 (7)	0.0388 (7)	0.0224 (6)	−0.0076 (5)	−0.0010 (5)	−0.0016 (5)
C6	0.0251 (5)	0.0286 (6)	0.0236 (5)	−0.0064 (4)	0.0095 (4)	0.0016 (4)
C7	0.0257 (6)	0.0252 (6)	0.0347 (6)	−0.0023 (4)	0.0143 (5)	0.0006 (4)
C8	0.0207 (5)	0.0302 (6)	0.0295 (6)	−0.0024 (4)	0.0049 (4)	0.0067 (5)
C9	0.0241 (5)	0.0252 (5)	0.0255 (5)	−0.0081 (4)	0.0073 (4)	−0.0012 (4)
C10	0.0229 (5)	0.0214 (5)	0.0287 (5)	−0.0040 (4)	0.0085 (4)	0.0035 (4)
C11	0.0456 (7)	0.0327 (7)	0.0274 (6)	−0.0083 (5)	0.0115 (5)	0.0013 (5)
C12	0.0804 (12)	0.0327 (8)	0.0472 (9)	0.0012 (8)	0.0135 (8)	0.0030 (6)
C13	0.0202 (5)	0.0248 (5)	0.0243 (5)	0.0029 (4)	0.0056 (4)	0.0009 (4)
C14	0.0251 (6)	0.0422 (7)	0.0344 (7)	0.0009 (5)	0.0125 (5)	0.0040 (5)
C15	0.0239 (6)	0.0536 (8)	0.0260 (6)	0.0080 (5)	0.0018 (5)	0.0013 (5)
C16	0.0336 (6)	0.0290 (6)	0.0376 (7)	−0.0114 (5)	0.0151 (5)	−0.0100 (5)
C17	0.0317 (6)	0.0356 (7)	0.0307 (6)	−0.0024 (5)	0.0006 (5)	−0.0136 (5)
C18	0.0449 (7)	0.0275 (6)	0.0219 (5)	0.0021 (5)	0.0112 (5)	−0.0057 (4)
C19	0.0324 (6)	0.0229 (5)	0.0304 (6)	0.0021 (4)	0.0128 (5)	−0.0038 (4)
C20	0.0398 (7)	0.0203 (5)	0.0272 (6)	−0.0017 (5)	0.0102 (5)	−0.0002 (4)
N1	0.0226 (4)	0.0200 (4)	0.0188 (4)	−0.0035 (3)	0.0040 (4)	0.0004 (3)
N2	0.0197 (4)	0.0225 (5)	0.0204 (4)	0.0011 (3)	0.0057 (4)	0.0004 (3)
O1	0.0328 (4)	0.0215 (4)	0.0257 (4)	−0.0057 (3)	0.0119 (3)	−0.0039 (3)
O2	0.0341 (4)	0.0281 (4)	0.0199 (4)	−0.0015 (3)	0.0071 (3)	0.0021 (3)
Ti1	0.02048 (11)	0.01844 (11)	0.01757 (11)	−0.00347 (7)	0.00591 (8)	−0.00093 (6)
Ti2	0.02010 (11)	0.02133 (11)	0.01558 (10)	0.00042 (7)	0.00478 (8)	−0.00092 (6)

### Geometric parameters (Å, º)

**Table:**

C1—C2	1.3133 (18)	C13—N2	1.4665 (14)
C1—O1	1.3443 (14)	C13—C14	1.5198 (16)
C1—H1	0.9500	C13—C15	1.5267 (16)
C2—H2A	0.9500	C13—H13	1.0000
C2—H2B	0.9500	C14—H14A	0.9800
C3—N1	1.4703 (14)	C14—H14B	0.9800
C3—C4	1.5124 (17)	C14—H14C	0.9800
C3—C5	1.5197 (17)	C15—H15A	0.9800
C3—H3	1.0000	C15—H15B	0.9800
C4—H4A	0.9800	C15—H15C	0.9800
C4—H4B	0.9800	C16—C20	1.4028 (18)
C4—H4C	0.9800	C16—C17	1.4118 (19)
C5—H5A	0.9800	C16—Ti2	2.3617 (12)
C5—H5B	0.9800	C16—H16	0.9500
C5—H5C	0.9800	C17—C18	1.3963 (19)
C6—C7	1.4039 (17)	C17—Ti2	2.3869 (12)
C6—C10	1.4155 (16)	C17—H17	0.9500
C6—Ti1	2.4096 (11)	C18—C19	1.3986 (18)
C6—H6	0.9500	C18—Ti2	2.4262 (11)
C7—C8	1.4093 (18)	C18—H18	0.9500
C7—Ti1	2.4140 (12)	C19—C20	1.4158 (16)
C7—H7	0.9500	C19—Ti2	2.4087 (11)
C8—C9	1.4071 (17)	C19—H19	0.9500
C8—Ti1	2.3878 (11)	C20—Ti2	2.3773 (12)
C8—H8	0.9500	C20—H20	0.9500
C9—C10	1.4094 (16)	N1—Ti1^i^	1.8296 (9)
C9—Ti1	2.3706 (11)	N1—Ti1	1.9972 (10)
C9—H9	0.9500	N2—Ti2^ii^	1.8864 (9)
C10—Ti1	2.3801 (11)	N2—Ti2	1.9398 (9)
C10—H10	0.9500	O1—Ti1	1.8834 (8)
C11—C12	1.315 (2)	O2—Ti2	1.8913 (8)
C11—O2	1.3395 (16)	Ti1—N1^i^	1.8296 (9)
C11—H11	0.9500	Ti1—Ti1^i^	2.7725 (4)
C12—H12A	0.9500	Ti2—N2^ii^	1.8864 (9)
C12—H12B	0.9500	Ti2—Ti2^ii^	2.8022 (4)
			
C2—C1—O1	124.77 (11)	C19—C18—H18	125.9
C2—C1—H1	117.6	Ti2—C18—H18	121.7
O1—C1—H1	117.6	C18—C19—C20	108.23 (11)
C1—C2—H2A	120.0	C18—C19—Ti2	73.87 (7)
C1—C2—H2B	120.0	C20—C19—Ti2	71.58 (7)
H2A—C2—H2B	120.0	C18—C19—H19	125.9
N1—C3—C4	109.44 (10)	C20—C19—H19	125.9
N1—C3—C5	112.22 (9)	Ti2—C19—H19	120.4
C4—C3—C5	111.57 (10)	C16—C20—C19	107.48 (11)
N1—C3—H3	107.8	C16—C20—Ti2	72.18 (7)
C4—C3—H3	107.8	C19—C20—Ti2	74.01 (7)
C5—C3—H3	107.8	C16—C20—H20	126.3
C3—C4—H4A	109.5	C19—C20—H20	126.3
C3—C4—H4B	109.5	Ti2—C20—H20	119.4
H4A—C4—H4B	109.5	C3—N1—Ti1^i^	151.07 (8)
C3—C4—H4C	109.5	C3—N1—Ti1	114.31 (7)
H4A—C4—H4C	109.5	Ti1^i^—N1—Ti1	92.75 (4)
H4B—C4—H4C	109.5	C13—N2—Ti2^ii^	133.70 (7)
C3—C5—H5A	109.5	C13—N2—Ti2	129.43 (7)
C3—C5—H5B	109.5	Ti2^ii^—N2—Ti2	94.16 (4)
H5A—C5—H5B	109.5	C1—O1—Ti1	131.09 (7)
C3—C5—H5C	109.5	C11—O2—Ti2	129.76 (8)
H5A—C5—H5C	109.5	N1^i^—Ti1—O1	106.79 (4)
H5B—C5—H5C	109.5	N1^i^—Ti1—N1	87.25 (4)
C7—C6—C10	107.71 (10)	O1—Ti1—N1	101.68 (4)
C7—C6—Ti1	73.25 (6)	N1^i^—Ti1—C9	93.65 (4)
C10—C6—Ti1	71.67 (6)	O1—Ti1—C9	142.01 (4)
C7—C6—H6	126.1	N1—Ti1—C9	111.16 (4)
C10—C6—H6	126.1	N1^i^—Ti1—C10	121.55 (4)
Ti1—C6—H6	120.7	O1—Ti1—C10	130.56 (4)
C6—C7—C8	108.34 (11)	N1—Ti1—C10	90.95 (4)
C6—C7—Ti1	72.91 (7)	C9—Ti1—C10	34.52 (4)
C8—C7—Ti1	71.91 (7)	N1^i^—Ti1—C8	97.76 (4)
C6—C7—H7	125.8	O1—Ti1—C8	109.60 (4)
C8—C7—H7	125.8	N1—Ti1—C8	145.17 (4)
Ti1—C7—H7	121.1	C9—Ti1—C8	34.40 (4)
C9—C8—C7	107.98 (10)	C10—Ti1—C8	57.08 (4)
C9—C8—Ti1	72.13 (6)	N1^i^—Ti1—C6	150.56 (4)
C7—C8—Ti1	73.96 (7)	O1—Ti1—C6	96.60 (4)
C9—C8—H8	126.0	N1—Ti1—C6	105.55 (4)
C7—C8—H8	126.0	C9—Ti1—C6	57.12 (4)
Ti1—C8—H8	119.7	C10—Ti1—C6	34.37 (4)
C8—C9—C10	107.96 (10)	C8—Ti1—C6	56.77 (4)
C8—C9—Ti1	73.47 (6)	N1^i^—Ti1—C7	129.22 (4)
C10—C9—Ti1	73.11 (6)	O1—Ti1—C7	85.81 (4)
C8—C9—H9	126.0	N1—Ti1—C7	139.30 (4)
C10—C9—H9	126.0	C9—Ti1—C7	56.86 (4)
Ti1—C9—H9	119.3	C10—Ti1—C7	56.70 (4)
C9—C10—C6	108.01 (10)	C8—Ti1—C7	34.13 (4)
C9—C10—Ti1	72.37 (6)	C6—Ti1—C7	33.84 (4)
C6—C10—Ti1	73.96 (6)	N1^i^—Ti1—Ti1^i^	46.02 (3)
C9—C10—H10	126.0	O1—Ti1—Ti1^i^	109.66 (3)
C6—C10—H10	126.0	N1—Ti1—Ti1^i^	41.24 (3)
Ti1—C10—H10	119.5	C9—Ti1—Ti1^i^	107.58 (3)
C12—C11—O2	124.91 (14)	C10—Ti1—Ti1^i^	110.93 (3)
C12—C11—H11	117.5	C8—Ti1—Ti1^i^	132.88 (3)
O2—C11—H11	117.5	C6—Ti1—Ti1^i^	140.16 (3)
C11—C12—H12A	120.0	C7—Ti1—Ti1^i^	164.44 (3)
C11—C12—H12B	120.0	N2^ii^—Ti2—O2	107.20 (4)
H12A—C12—H12B	120.0	N2^ii^—Ti2—N2	85.84 (4)
N2—C13—C14	109.58 (9)	O2—Ti2—N2	103.11 (4)
N2—C13—C15	113.45 (9)	N2^ii^—Ti2—C16	117.65 (4)
C14—C13—C15	110.07 (10)	O2—Ti2—C16	131.96 (4)
N2—C13—H13	107.9	N2—Ti2—C16	96.63 (4)
C14—C13—H13	107.9	N2^ii^—Ti2—C20	88.59 (4)
C15—C13—H13	107.9	O2—Ti2—C20	140.36 (4)
C13—C14—H14A	109.5	N2—Ti2—C20	114.35 (4)
C13—C14—H14B	109.5	C16—Ti2—C20	34.43 (5)
H14A—C14—H14B	109.5	N2^ii^—Ti2—C17	145.09 (4)
C13—C14—H14C	109.5	O2—Ti2—C17	97.53 (4)
H14A—C14—H14C	109.5	N2—Ti2—C17	112.49 (4)
H14B—C14—H14C	109.5	C16—Ti2—C17	34.59 (5)
C13—C15—H15A	109.5	C20—Ti2—C17	57.08 (5)
C13—C15—H15B	109.5	N2^ii^—Ti2—C19	92.34 (4)
H15A—C15—H15B	109.5	O2—Ti2—C19	107.19 (4)
C13—C15—H15C	109.5	N2—Ti2—C19	148.75 (4)
H15A—C15—H15C	109.5	C16—Ti2—C19	56.89 (4)
H15B—C15—H15C	109.5	C20—Ti2—C19	34.41 (4)
C20—C16—C17	107.95 (11)	C17—Ti2—C19	56.31 (4)
C20—C16—Ti2	73.39 (7)	N2^ii^—Ti2—C18	123.60 (4)
C17—C16—Ti2	73.68 (7)	O2—Ti2—C18	85.03 (4)
C20—C16—H16	126.0	N2—Ti2—C18	146.12 (4)
C17—C16—H16	126.0	C16—Ti2—C18	56.71 (4)
Ti2—C16—H16	118.8	C20—Ti2—C18	56.67 (4)
C18—C17—C16	108.20 (11)	C17—Ti2—C18	33.72 (5)
C18—C17—Ti2	74.68 (7)	C19—Ti2—C18	33.63 (4)
C16—C17—Ti2	71.73 (7)	N2^ii^—Ti2—Ti2^ii^	43.66 (3)
C18—C17—H17	125.9	O2—Ti2—Ti2^ii^	110.86 (3)
C16—C17—H17	125.9	N2—Ti2—Ti2^ii^	42.18 (3)
Ti2—C17—H17	119.5	C16—Ti2—Ti2^ii^	113.10 (3)
C17—C18—C19	108.13 (11)	C20—Ti2—Ti2^ii^	105.60 (3)
C17—C18—Ti2	71.60 (7)	C17—Ti2—Ti2^ii^	144.77 (4)
C19—C18—Ti2	72.50 (7)	C19—Ti2—Ti2^ii^	128.26 (3)
C17—C18—H18	125.9	C18—Ti2—Ti2^ii^	161.30 (3)

Symmetry codes: (i) −*x*+2, −*y*+1, −*z*; (ii) −*x*+1, −*y*, −*z*.
